# Scour Monitoring System for Subsea Pipeline Based on Active Thermometry: Numerical and Experimental Studies

**DOI:** 10.3390/s130201490

**Published:** 2013-01-24

**Authors:** Xuefeng Zhao, Weijie Li, Gangbing Song, Zuo Zhu, Jun Du

**Affiliations:** 1 School of Civil Engineering, Dalian University of Technology, Dalian 116024, China;E-Mails: jack199008@gmail.com (W.L.); 88614@mail.dlut.edu.cn (Z.Z.); dagongdj@mail.dlut.edu.cn (J.D.); 2 Department of Mechanical Engineering, University of Houston, Houston, TX 77204-4006, USA; E-Mail: gsong@uh.edu

**Keywords:** Brillouin optical fiber sensor, scour monitoring, subsea pipeline, active thermometry, numerical simulation

## Abstract

A scour monitoring system for subsea pipeline based on active thermometry is proposed in this paper. The temperature reading of the proposed system is based on a distributed Brillouin optical fiber sensing technique. A thermal cable acts as the main component of the system, which consists of a heating belt, armored optical fibers and heat-shrinkable tubes which run parallel to the pipeline. The scour-induced free span can be monitored through different heat transfer behaviors of in-water and in-sediment scenarios during heating and cooling processes. Two sets of experiments, including exposing different lengths of the upper surface of the pipeline to water and creating free spans of various lengths, were carried out in laboratory. In both cases, the scour condition was immediately detected by the proposed monitoring system, which confirmed the system is robust and very sensitive. Numerical study of the method was also investigated by using the finite element method (FEM) with ANSYS, resulting in reasonable agreement with the test data. This brand new system provides a promising, low cost, highly precise and flexible approach for scour monitoring of subsea pipelines.

## 1. Introduction

With the increasing demand for energy all over the World, oil, natural gas, and its processing facilities have become important assets for many countries. One such asset are the subsea pipelines used to transfer oil and natural gas. Typically, subsea pipelines are buried under the seabed. However, the sub-sea currents around the pipelines will gradually remove the aerodynamic cover and anchoring foundation of the pipelines and induce a local disturbance in the wave/current flow. When the current flow is obstructed by the pipeline, a pressure gradient is established between the upstream and downstream sides of the obstruction. If the sea-bottom is permeable, such a pressure gradient drives a seepage flow between the pipe and the seabed. This seepage flow tends to drag sand particles and, if the flow is sufficiently large, is able to wash away the sand beneath the pipeline, giving rise to the onset of scouring underneath the pipe [1]. If the scour is only locally overcritical, it will lead to a free span. Once the free span length exceeds the allowable length, the pipelines would be subjected to high stress and even vortex-induced resonance if their natural frequencies were approximately equivalent to the vortex shedding frequencies, leading to potential structural failure. Thousands of accidents have been reported proving that the consequence of pipeline failure is both economically and environmentally disastrous [2,3]. Therefore, having a reliable, real-time and robust scour monitoring system for subsea pipelines can significantly help in inspecting and maintaining them.

In recent years, a number of methods have been proposed to monitor scour of subsea pipelines. In 2003, Jin *et al.* [4] introduced a basic real-time monitoring system for long distance submarine pipelines. Distributed optical fiber sensors were deployed to monitor the strain along the pipeline. The introduced system functions as an auto-alarm and a damage locator. Likewise, Jin and Shao introduced a practical algorithm for data analysis and demonstrated a practical application of the system on the submarine pipeline in Bohai Sea, China [5]. In 2011, Bao and Hao developed an integrated autoregressive moving average (ARMA) model algorithm for the structural health monitoring (SHM) of offshore pipelines [6]. Both numerical simulations and laboratory tests proved that the method is sensitive to pipeline damage. The methods mentioned above concentrate on indirectly measuring the free-spanning vibration. However, these kinds of methods are feasible only if the vortex-induced vibration is large enough to be detected and larger than the noise and measurement error. Furthermore, these scour monitoring systems are constrained by the construction limitations. During pipeline construction, the pipes are welded one by one on a ship before being placed on the sea bottom. Installing the distributed fiber optical strain sensors presents a great challenge for commercial practical application. Unlike the conventional scour monitoring methods, in this paper, we propose a scour monitoring system based on active thermometry. Active thermometry is an effective way to measure thermal parameters, namely thermal resistivity, thermal diffusivity, specific heat and soil water content [7–11]. Previous research shows that active thermometry, implemented with optical fiber temperature sensors, has enormous potential in the field structural health monitoring [12,13].

To supply a practical solution which is applicable along the entire length of a subsea pipeline, we employ the distributed Brillouin optical fiber sensing technique, which has been widely used in many fields in recent years. Optical fiber sensing techniques offers many unique advantages such as small size, high sensitivity, resistance to electromagnetic interference, low signal decay, accessibility to harsh environment and long-term measurement reliability. With these advantages, proper packaged optical fibers are capable of detecting the scour of subsea pipelines even when surrounded by complex and turbulent hydrodynamic and geographic environments. Due to the large pipeline span that must be monitored and the linear nature of pipelines, the distributed optical fiber sensing technique offers significant advantages by providing distributed strain and temperature data along the sensing optical fiber in both space and time domain. Fully distributed optical fiber sensor uses optical time domain reflectometry technology to measure the strain and temperature along the optical fiber that acts at the same time as a distributed transducer and optical channel, thereby resulting in a relatively low cost compared to other distributed sensing techniques.

The main component of the proposed subsea pipeline scour monitoring system is a thermal cable bonded to the pipelines. Therefore, when it comes to scour monitoring, the thermal cable shares the same ambient conditions with the pipelines. The heat transfer behaviors between sediment and water are different. As a result, it is possible to identify the ambient media as sediment or water by analyzing temperature information during the heating and cooling processes. Once the ambient media along the pipeline is identified as either sediment or water, the water-sediment interfaces are detected and the scour induced exposure as well as the free span of the pipeline are obtained. This is the key technique of the proposed scour monitoring system. The proposed monitoring system can be broken into three parts as follows: the thermal cable, the data acquisition unit (DAU) and the data processing unit (DPU). The thermal cables, which run parallel to the pipelines, consist of a heating belt, distributed optical fiber temperature sensors and packaging elements. The system first uses the heating belt to generate heating along the thermal cable while the distributed temperature sensors concurrently measure the temperature along the thermal cable. The DPU then extracts the information and analyzes the acquired data to identify whether the thermal cable is exposed to water or buried in sediment, thus determining the scour condition of the pipeline. Rather than being mounted on the surface of the pipes, the armored cable can be installed in the vicinity of the pipes, suggesting that the pipes and the cables will be exposed to water at the same time and the same location when scour occurs. The proposed scour monitoring system in this paper can make the installation of distributed fiber optical temperature sensors along the pipe relatively simple, which can avoid many construction constraints and make it highly practical.

In an earlier study [14], the feasibility of the proposed method was examined. However, the heat transfer behaviors for a linear heat source in water and sediment have not been fully understood. It may hard to understand the development of the scour condition of the pipeline by just installing one thermal cable in the pipeline. Moreover, when it comes to field applications, it will face problems such as the power consumption of the constant-temperature heating belt and length limitation of the heating belt. Further investigations are presented in this study.

## 2. Theory Background

### 2.1. Principle of Brillouin Optical Fiber Sensing Technique

The Brillouin optical fiber sensing technique is based on the Brillouin scattering phenomena. An optical fiber is utilized both as a distributed transducer and as an optical channel. When a short light pulse is launched into and transmitted along the fiber, the frequency of the Brillouin backscattering light can be measured at the same end. The time interval between sending the pulse and arrival of the backscattered light provides the spatial information, while the frequency of the backscattering light provides information of the temperature or strain distributed along the optical fiber. The backscattered Brillouin frequency is shifted from the incident light frequency because of the temperature variation and strain variation along the optical fiber. The Brillouin frequency shift has a linear dependence on the applied longitudinal strain and the temperature variation that can be written as [15]:

(1) $$\{\begin{array}{c} \upsilon_{B}(ɛ)=\upsilon_{B}(0)+C_{S}ɛ\\ \upsilon_{B}(T)=\upsilon_{B}(T_{0})+C_{t}(T-T_{0}) \end{array}$$

where *υ_B_*(*ε*), *υ_B_*(0), *υ_B_*(*T*) and *υ_B_*(*T*_0_) are frequency shifts under strain *ε* and 0, and under temperature *T* and *T*_0_, respectively.*C_S_* and *C_t_* are the strain and temperature coefficients, respectively. In the case of pure temperature variations, there will be no strain variations, so the relation can be expressed as:

(2) $$\Delta \upsilon_{B}=C_{t}\Delta T$$

where Δ*υ_B_* = Δ*υ_B_*(*T*) − Δ*υ_B_*(*T*_0_) is defined as frequency shift and Δ*T* = *T* − *T*_0_ is defined as the excess temperature.

### 2.2. Heat Transfer Behaviors in Solid and Liquid

There are two means of heat transfer in solids and in liquids, namely conduction and convection. For sections buried in sediment, heat transfer is by means of conduction, and the problem can be idealized as an infinite line source in an infinite, homogeneous, isotropic medium. By using the “Transient heat method” [16], for large value of *t* (*t* ≫ *r*^2^/(4*α*) and *t* − *t*_1_ ≫ *r*^2^/(4*α*), respectively), excess temperature can be expressed as:

(3) $$\{\begin{array}{c} \Delta T=\frac{q}{4\pi \lambda }\left(\text{In}t+\text{In}\frac{4\alpha }{r^{2}}-\gamma \right)t\leq t_{1}\\ \Delta T=\frac{q}{4\pi \lambda }\text{In}\frac{t}{t-t_{1}}t>t_{1} \end{array}$$

where Δ*T* is the excess temperature, Δ*T* = *T* − *T*_0_; *T*_0_ is the initial temperature; *γ* is Euler's constant (*γ* = 0.5772); *q* is the quantity of heat released per unit length of the line source during heating, which starts at *t* = 0 and stops at *t* = *t*_1_; *α* the thermal diffusivity of the solid (*α* = *λ*/*ρc*); *λ*, *ρ* and *c* are the thermal conductivity, the density and the specific heat of the solid, respectively; and *r* is the distance to line source.

For sections surrounded by water, heat transfer is by means of convection, in this study, the thermal resistance of the thermal cable can be neglected due to the small cross-section area of the thermal cable. The lumped parameter method is adopted by assuming the inner temperature is uniform within any given cross section of the thermal cable. The problem is simplified to:

(4) $$\{\begin{array}{c} \rho cV\frac{\partial T}{\partial t}=q-Ah(T-T_{0})t\leq t_{1}\\ \rho cV\frac{\partial T}{\partial t}=-Ah(T-T_{0})t>t_{1}\\ T=T_{0}\;t=0 \end{array}$$

where *h* is the convective heat transfer coefficient; *ρ* and c are the density and the specific heat, and *A* and *V* are the convective area and volume per unit length of the sensor, respectively. The solution is:

(5) $$\{\begin{array}{c} \Delta T=\frac{q}{hA}(1-exp(-t/\tau_{c}))t\leq t_{1}\\ \Delta T=(T(t_{1})-T_{0})\cdot \exp(-(t-t_{1})/\tau_{c}))t>t_{1} \end{array}$$

where the time constant *τ_c_* = *ρcV*/*hA*.

## 3. Numerical Study

To verify the feasibility of the proposed active thermometry method for scour monitoring of subsea pipelines, the heat transfer behaviors among the thermal cable, sediment and water are obtained through numerical simulation and analysis.

### 3.1. FE Modeling of the Scour Monitoring System for Subsea Pipeline

**Figure 1.**
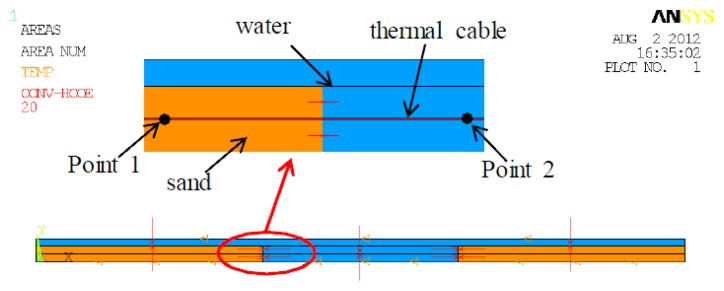
Finite element model of the pipeline system.

In the present study, the FE model of a scour monitoring system was established using the commercial FE software ANSYS (ANSYS, Inc, Canonsburg, PA, USA). The experimental model of the scour monitoring system, which will be detailed in Section 4, was taken as the prototype of FE modeling. For convenience and the minimization of computing cost, the scour monitoring system was transformed into a two-dimensional model. The free span was in the middle of the model, with a length of 6 m, and sand was on the left and the right, respectively. The water, kept a constant depth of 0.7 m, was considered stagnant in the numerical simulation. The overview and details of the finite element model are shown in Figure 1.

For the transient analysis, a 4-node, 2D thermal element PLANE55 was employed in modeling the system. Because of the complicated hydrodynamic and geomorphic conditions, it is challenging to accurately obtain all of the thermal parameters of the surrounding medium along the pipeline. The measurement of thermal parameters is beyond the scope of this study, at least for the moment. Therefore, empirical parameters for thermal properties were used in this study. Material parameters for water, sand and copper are listed in Table 1. Of particular note are sand and copper. Sand was the sediment in this study and copper was the main component of the thermal cable. Thus, sand and copper were used to represent sediment and thermal cable, respectively.

The uniform temperature of the whole model was set to 20 °C (room temperature) and was considered constant. The left and right lines of the model were considered insulated, indicating no loads were applied on them. The ambient temperature, identical to room temperature, valued at 20 °C, was applied to the top and bottom lines. Convection exists along the interfaces between water and sand as well as between water and the thermal cable, whose film coefficient varies from 20 W/m^2^ · °C to 100 W/m^2^ · °*C* when there is natural convection. The convective film coefficient was set to 20 W/m^2^ · °*C* in this study. In order to simulate the heat generated within the constant-power thermal cable, heat generation rates (HGEN) was applied to thermal cable in the FE model. Specifically, the power of the thermal cable was 15 W/m, divided by the cross-section (0.009 m × 0.006 m), producing an HGEN of 277,778 W/m^3^. After all the loads were imposed to the FE model, simulation of the model in ANSYS was executed for a total of five hours, three hours for heating and two hours for cooling.

### 3.2. Simulation of Heat Transfer Behaviors of the Scour Monitoring System

**Figure 2.**
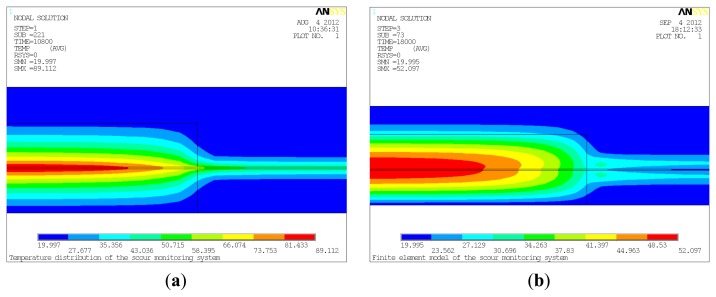
(**a**) Temperature distribution at t = 10,800 s. (**b**) Temperature distribution at t = 18,000 s.

To simulate the heating process of three hours, in this case, HGEN was imposed on the thermal cable for three hours. In Figure 2(a), the temperature distribution of the pipeline system at t = 10,800 s is partially shown. The highest temperature within the system was in the sand which was about 89.112 °C. In water, the temperature on the edge of the thermal cable was about 47.491 °C, which was much lower than that in sand. It can be seen that temperature along the thermal cable increased from water to sand, making the interface between water and sand identifiable. It should be noted that the heat conducted through the sand reached a wider range in comparison with convection through water. The reason for this is that heat dissipates more easily through convection via water than conduction via sand. While heat dissipates gradually through conduction in sand, heat can be directly carried away by water.

To simulate the two hour cooling process, the simulation was restarted after removing the HGEN in the thermal cable. The HGEN was set to 0 W/m^3^, while boundary conditions and other loads remained the same. The temperature distribution of the pipeline system after cooling for one hour is shown in Figure 2(b). Similar to the temperature distribution at t = 10,800 s, the temperature in the sand along the thermal cable was the highest at 52.097 °C; while in water, the temperature was approximately 23.060 °C. Other characteristics were approximately the same as that at t = 10,800 s.

**Figure 3.**
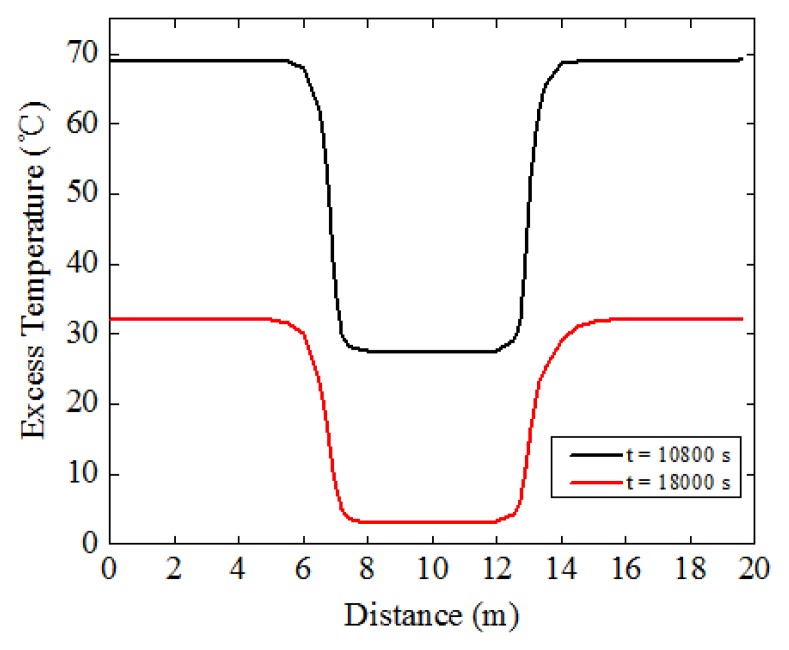
Variation of excess temperature with distance along the thermal cable; at t = 10,800 s and at t = 18,000 s.

Figure 3 shows the variation of excess temperature with distance along the thermal cable, at t = 10,800 s and t = 18,000 s. It can be clearly seen that, for both cases, temperature in the sand was higher than that in water while remaining constant in general. However, near the interfaces between sand and water, the temperature varied sufficiently enough to distinguish the interfaces. In sand, the overall temperature was higher, while in water, it was lower. The temperature gap can be used to identify the free span length. The actual free span length is 6 m, from 7 m to 13 m, as shown in Figure 1. In Figure 3, it is evident that the first interface between sand and water was between 6 m and 8 m along the thermal cable, and the second interface was between 12 m and 14 m. To be prudent, it can be said that the predicted free span in this case was between 6 m and 14 m, containing the actual free spans ranging from 7 m to 13 m. Note that there exists one meter deviation by taking safety into account in the numerical study, but considering the actual pipeline can be hundreds of kilometers in length, this level of deviation is negligible.

**Figure 4.**
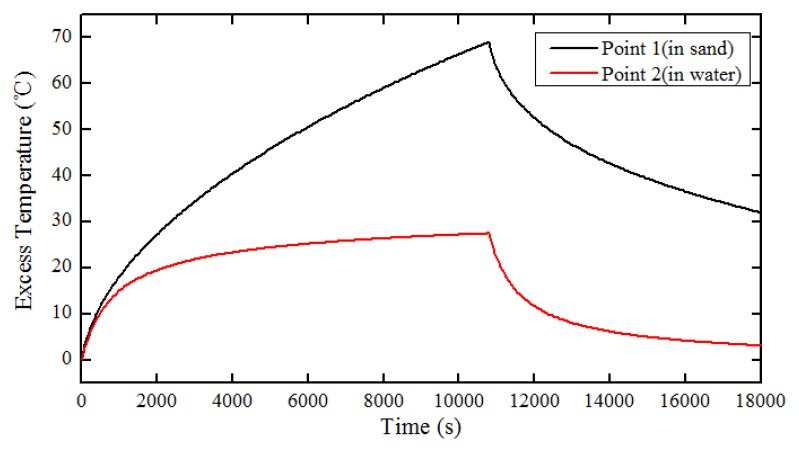
Variation of excess temperature with time for Point 1 and Point 2.

To further investigate the heat transfer behaviors of the scour monitoring system, two points were taken from the FE model as a demonstration. One point, marked Point 1, is located in the sand along the thermal cable; the other, marked Point 2, is located in water along the thermal cable as shown in Figure 1. Variation of excess temperature with time for Point 1 and Point 2 is shown in Figure 4. As can be seen, during the first 500 s, the excess temperatures for both Point 1 and Point 2 rapidly increased concurrently, overlapping in general. After that, the value of excess temperature for Point 1 increased gradually in the heating process. However, the value of temperature for Point 2 began to level off and experienced little change until cooling. Consequently, the temperature gap between Points 1 and 2 increased during the heating process, making them distinguishable. In the cooling process, the temperature curves for both Point 1 and Point 2 decreased exponentially. The value of temperature for Point 2 swiftly decreased and approaches the initial room temperature assymptotically. According to Equations (3) and (5), excess temperatures both in the sand and in the water will rise quickly during heating process. Subsequently, the temperature in the water will begin to stabilize and while the temperature in the sand will continue to rise at a decaying rate. The temperature in the sand exceeds those in water just in several minutes and enlarges the temperature gap slowly. In the cooling process, excess temperature in water decline exponentially and finally return to zero while those in sand decrease at a quickly decaying rate. Therefore, the temperature is higher in sand than that in water. Based upon the characteristics described above, the in-water and in-sand scenarios can be identified, the interfaces between in-water and in-sand scenarios are recognizable, and then the free span is detected.

Based on the results obtained from numerical simulation of the pipeline system, heat transfer behaviors among the thermal cable, sand and water were analyzed. The results have confirmed that numerical simulation coincides with the theoretical studies, which confirms the feasibility of the proposed active thermometry method for scour monitoring of subsea pipelines. However, near the interfaces between sand and water, both conduction and convection occurred (Figure 2), therefore the simulation becomes a fluid-solid couple problem, making it difficult to accurately locate the position of the interfaces. Thus, it calls for further study to fully understand the heat transfer behaviors in the interfaces to determine the precise location of it. Even though the results show good agreement with the theoretical studies, the water was considered stagnant which is inconsistent with the actual environment. The water is dynamic in most cases, and greater flow velocity leads to larger convective film coefficient thereby dissipating the heat at a faster rate. By taking these effects into consideration, further simulation should be carried out using computational fluid dynamics (CFD) in ANSYS/FLOTRAN to get the more satisfying results, but the limited computer resources should be weighed.

## 4. Experiment

### 4.1. Set-up of the Scour Monitoring System

**Figure 5.**
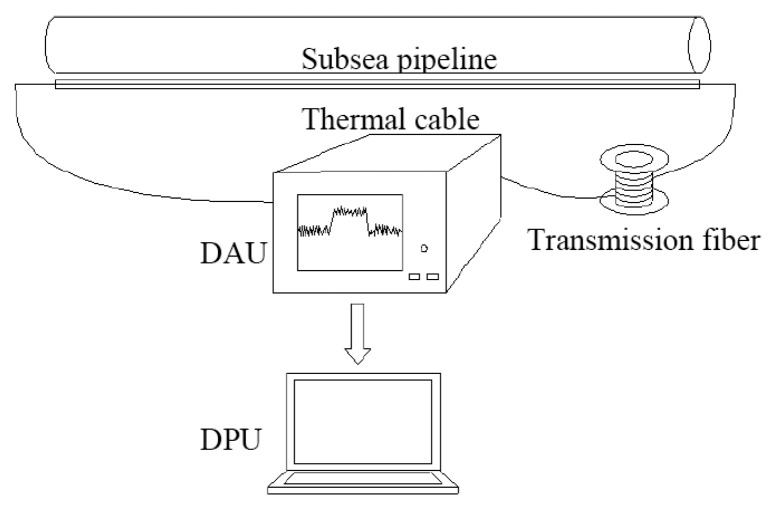
Schematic diagram of the scour monitoring system.

**Figure 6.**
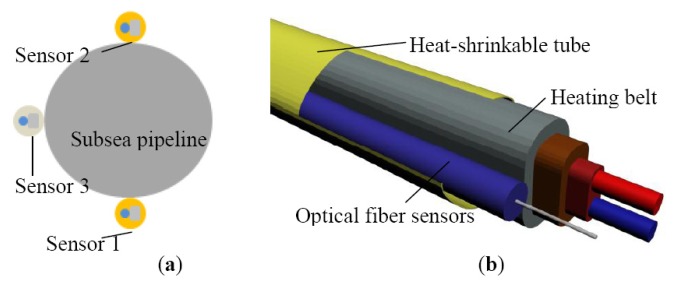
(**a**) Scheme of thermal cable placement with respect to the pipe section. (**b**) Illustration of the thermal cable.

The scour monitoring system was composed of several thermal cables, data acquisition unit (DAU) and data processing unit (DPU), as shown in Figure 5. The thermal cable was made up of a heating belt, armored optical fibers, and heat-shrinkable tubes, as shown in Figure 5. Two kinds of thermal cables were designed in this study. One of the cables was a constant-power thermal cable with a constant-power heating belt equipped inside, and the other was constant-temperature thermal cable with a constant-temperature heating belt equipped inside. The constant-power heating belt was 21 m in length with a cross-section dimension of 9 mm × 6 mm, whose maximum output power was 15 W/m. The power source for the heating belt was supplied by an explosion-proof temperature controller, thus, the heating temperature was controllable, ranging from 0 °C to 120 °C. The temperature was set to 80 °C in the experiment. The constant-temperature heating belt was 21 m in length and of a cross-section of 2 mm × 10 mm, whose maximum surface temperature was 110 °C. The armored optical fibers were attached to the heating belt using insulating tape. To protect them from water, they were carefully encapsulated in heat-shrinkable tubes. In the present study, there were three thermal cables in total, and were positioned in the following configuration: constant-power thermal cables were put on the upper surface and lower surface of the pipeline, and a constant-temperature thermal cable was put on the left side of the pipeline as illustrated in Figure 6. For temperature measurement, we adopted the distributed Brillouin fiber optic sensing technique. The Brillouin Optical Time-Domain Analysis (BOTDA) analyzer along with the DiTest^™^ STA100/200 Series-Fiber Optic Distributed Temperature and Strain Analyzer, acting as the DAU, was used to measure temperature directly. In order to measure the temperature of all the three thermal cables at the same time and on the same channel, the optical fibers were connected one by one, indicating that the end of one fiber was connected to the start of another fiber. The fiber circuit began with the optical fiber sensors in the bottom (named Sensor 1), followed by the one on the top (named Sensor 2), then to the one on the left (named Sensor 3), as shown in Figure 6. Finally, they were connected to the BOTDA analyzer. Specifications for the analyzer are listed in Table 2. The data was exported to a laptop to be stored and analyzed, which served as the DPU.

### 4.2. Scour Monitoring System Experiments

**Figure 7.**
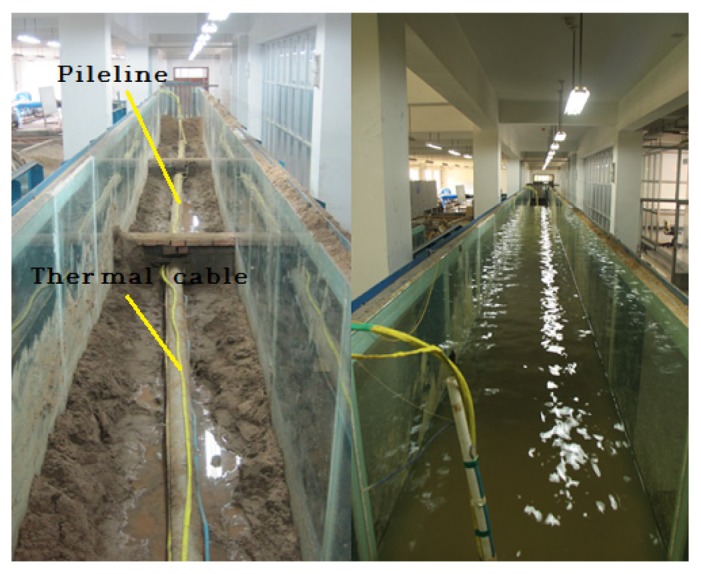
Experimental set-up: the thermal cables were placed in the vicinity of the pipeline (**left**) and the system operated in running water environment (**right**).

To validate the scour monitoring system, experiments were conducted in the laboratory. A 21 m long section was partitioned from a 48 m long by 1 m wide and 1.5 m high indoor experimental flume whose ends were blocked by brick walls. There was a water inlet and a water outlet in each end of the flume. The brick walls were 0.6 m in height and could let water pass through. A controllable water cycle was created by using a pump so that the experiments were conducted in a running water environment. Three 6 m long steel tubes were welded end-to-end to form an 18 m long steel tube. Each tube had a diameter of 100 mm and a thickness of 2.5 mm. The ends and joints of the welded tube were shielded from water. The tube was subsequently placed in the middle of the separated flume section, with a distance of 20 cm from the bottom, acting as a subsea pipeline, as shown in Figure 7. The thermal cables ran parallel to the tube with each end of the cable extending 1.5 m from the end of the tube. The cable was secured to the tube by using iron wires with 0.5 m spacing. The selected 21 m flume was further divided into three sections by using shorter brick walls. The two outer sections were roughly 7 m in length and the middle section was 6 m in length. The three sections were filled with sand of 0.5 m high which acted as the sediment.

Prior to performing the experiments, the sand was fully saturated, that is to say, water was continuously added to the flume with a constant level of 0.7 m for 2 hours. After that, experiments were conducted as follows: First, the analyzer was activated to scan the optical fiber sensors for 6 minutes with the aim of acquiring initial temperature of the pipeline system. Second, the heating belt was connected to the power supply for 3 hours in order to generate heat. Lastly, after the 3 hours of heating, the heating belt was disconnected to allow a cool down of 2 hours before turning on the analyzer. The room temperature was recorded before performing every experiment.

**Figure 8.**
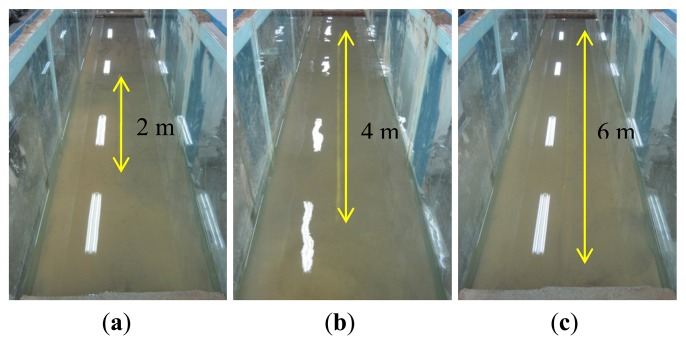
(**a**) Exposure of the upper surface of the pipeline with a length of 2 m. (**b**) Exposure of the upper surface of the pipeline with a length of 4 m. (**c**) Exposure of the upper surface of the pipeline with a length of 6 m.

**Figure 9.**
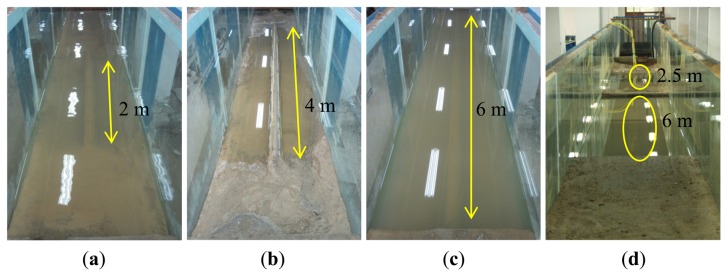
(**a**) Free span with a length of 2 m. (**b**) Free span with a length of 4 m. (**c**) Free span with a length of 6 m. (**d**) Test of two free spans (6 m + 2.5 m).

The experiments were divided into two parts. To simulate the early stage of subsea pipeline scour, the first experiment involved exposing the upper surface of the pipelines to water by taking into account the variation of the exposure length, including 2 m, 4 m and 6 m, as shown in Figure 8. The exposure conditions were then detected by the optical fiber sensors on the upper surface of the pipeline. The second experiment was conducted afterwards, which was to simulate the scour-induced free span of subsea pipeline by digging out the sediment. Likewise, the free span length was varied, including 2 m, 4 m and 6 m (Figure 9), and the free span conditions were detected by all the three optical fiber sensors. In addition, two free spans (2.5 m and 6 m) were also created to test the scour monitoring system, as shown in Figure 9. The measurements were repeated three times.

## 5. Results and Discussion

### 5.1. Results of Upper Surface Exposure Experiments

**Figure 10.**
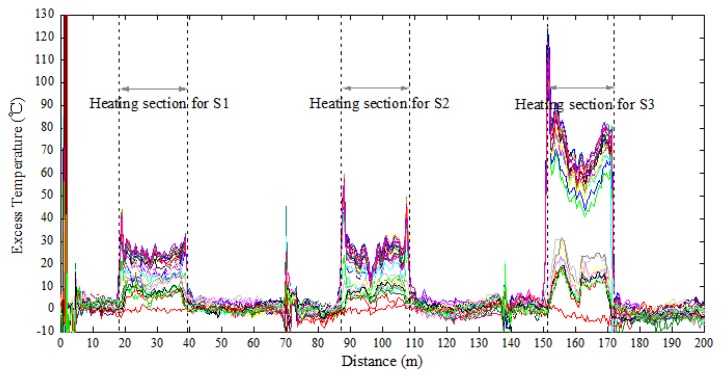
Location of the heating sections.

For the sake of saving computer storage, only the first 200 m of the fiber circuit was scanned. As can be seen from Figure 10, after heating for some time, the heated sections were noticeable, with each section being roughly 21 m in length. Each curve represents the sampling data of one scan and the excess temperature can be obtained by subtracting the corresponding values before heating. The working section for each sensor was approximately 18 m, Sensor 1 was located from 20.35 m to 37.44 m, Sensor 2 from 88.31 m to 106.62 m, and Sensor 3 from 153.01 m to 171.33 m.

**Figure 11.**
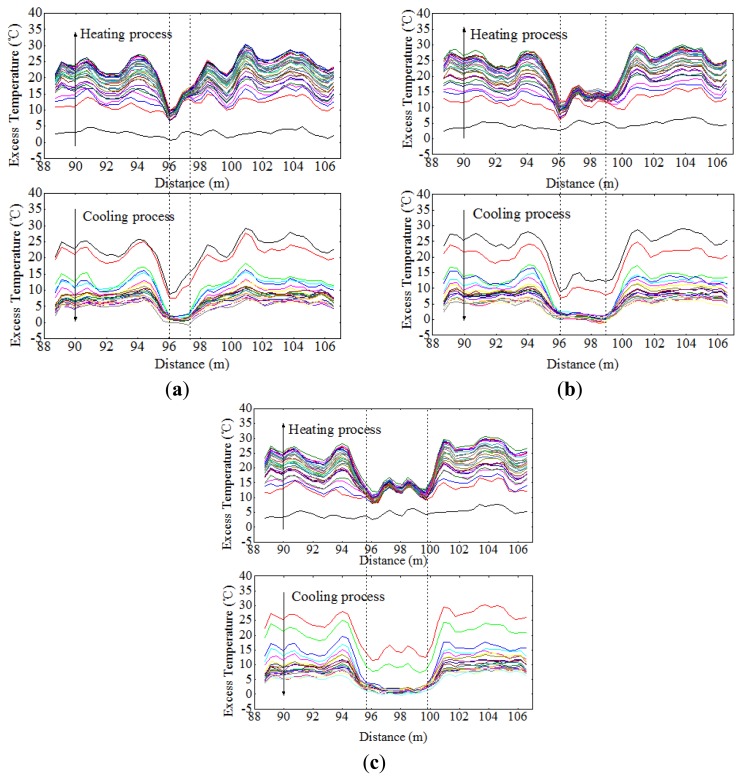
(**a**) Excess temperature curves of Sensor 2 under the condition that the upper surface of the pipeline exposed 2 m to water. (**b**) Excess temperature curves of Sensor 2 under the condition that the upper surface of the pipeline exposed 4 m to water. (**c**) Excess temperature curves of Sensor 2 under the condition that the upper surface of the pipeline exposed 6 m to water.

**Figure 12.**
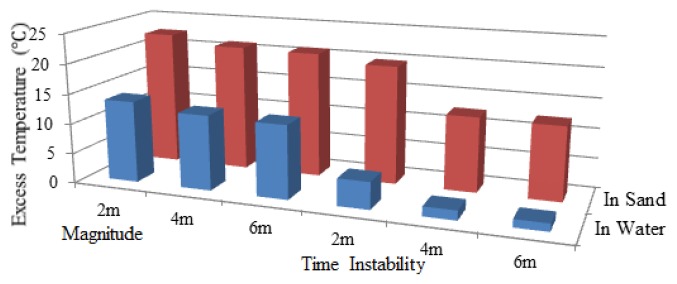
Two features for Sensor 2 under the condition that the upper surface of the pipeline exposed to water.

Since the scour was first monitored by Sensor 2, the working section of Sensor 2 was taken for analysis. Excess temperature data curves of Sensor 2 for water exposure of pipeline surface with the lengths of 2 m, 4 m and 6 m are shown in Figure 11. As can be seen from the figures, the section that was exposed to water can be identified by paying special attention to the excess temperature curves. The magnitudes for in sand and in water scenarios are different. The section exposed to water showed lower excess temperature than sections buried in sand. An apparent excess temperature gap of about 10 °C was quantified between these two scenarios by calculating the average excess temperature of all sampling points during the heating process. Second, the time instability for the two scenarios is obtained by calculating the variance with time during heating process. The excess temperature in the exposed section were more stable as a function of time and more intensive in both heating and cooling process, while the curves in buried sections are scattered along the vertical axis. The two features mentioned above contributed to identify the sections that exposed to water flow, as shown in Figure 12.

By paying close attention to the cooling process in Figure 11(a), there exists a set of intensive horizontal curves from 96.04 m to 97.26 m resulting from the corresponding section of the thermal cable being totally exposed to water. According to the Brillouin optical fiber sensing technique, the signal of a sampling point on the optical fiber is not the signal of the specific sampling point, but the average signal within a certain range relating to that point. This range is the spatial resolution. Consequently, when the identified section agrees with the features of the temperature change of the in-water scenario, it does not mean that this section is an entirely exposed section, but instead indicates that the locations within the resolution ranges of all the sampling points that yield similar data of this section are all immersed in water. To obtain the exposure length monitored by the system, the total exposed length (97.26 − 96.04 = 1.22 m) should be added to the resolution length of the optical fiber (1 m). Thus, the identified exposure length is 2.22 m, which is a difference of 0.22 m with experimental setup, as listed in Table 3. In the same way, those for exposure of 4 m and 6 m as well as error analysis are also listed in Table 3. As can be seen, the error rate decreased as the exposure length increased. The fact that the subsea pipelines are over hundreds of kilometers, this level of precision is more than adequate.

In this case, it is hard to determine the exposure length simply by analyzing the data from the heating process, but the data can serve as a good reference. By combining the data from the heating process with the cooling process, a convincing result can be obtained. In practice, there are a number of departures from the ideal situation. For example, the formation of air gaps within the thermal cable and the uneven sediment compactness will affect the uniformity of the excess temperature curves, making the detection of exposure length difficult.

### 5.2. Results of Free Span Experiments

Once the free span problem occurs, it can be immediately detected by all the optical fiber sensors. In free span experiments, Sensor 2 works the same as the exposure experiments, so it is needless to demonstrate it again. We thus focus on discussing the results acquired from Sensor 1 and Sensor 3.

**Figure 13.**
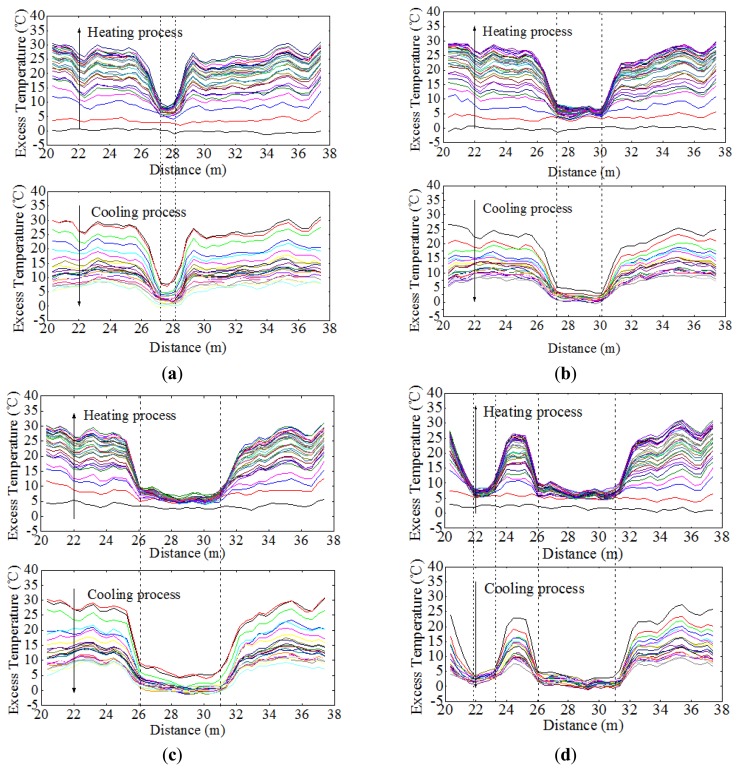
(**a**) Excess temperature curves of Sensor 1 for free span in 2 m. (**b**) Excess temperature curves of Sensor 1 for free span in 4 m. (**c**) Excess temperature curves of Sensor 1 for free span in 6 m. (**d**) Excess temperature curves of Sensor 1 for two free span test.

**Figure 14.**
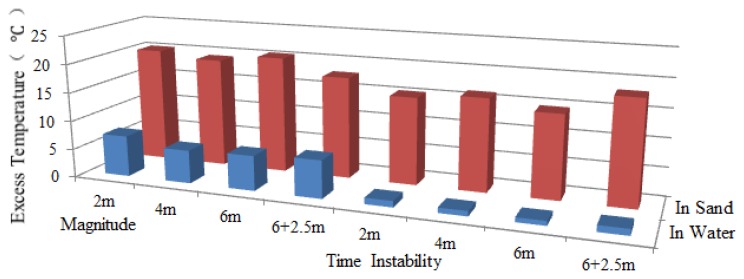
Two features for Sensor 1 under free spanning condition.

**Figure 15.**
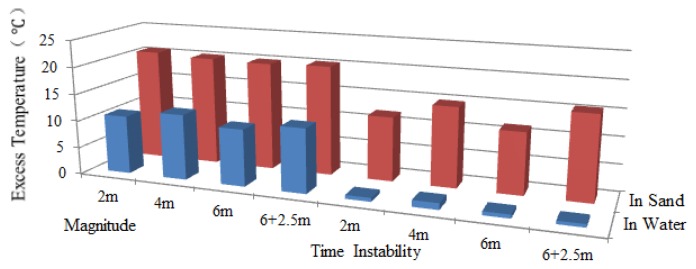
Two features for Sensor 2 under free spanning condition.

**Figure 16.**
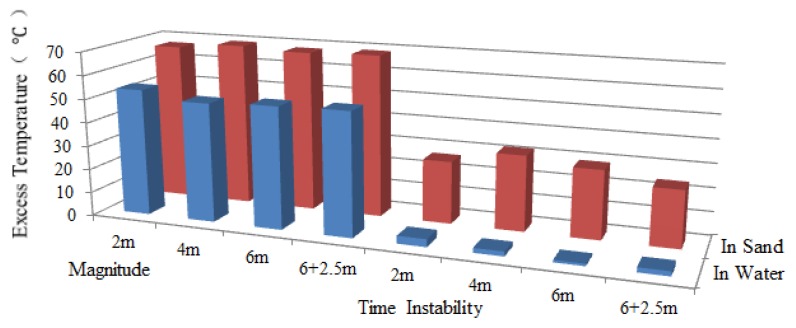
Two features for Sensor 3 under free spanning condition.

By adopting the analyzing method discussed above, when the pipeline had a span of 2 m, data acquired from Sensor 1 indicated that section entirely surrounded in water was from 27.27 m to 28.08 m, as shown in Figure 13(a). Likewise, the detected free span length should be added by the spatial resolution, which was 1.81 m with a difference value of 0.19 m. And the same went for those free-spanned with 4 m long and 6 m long. As a general rule, a pipeline has multiple instances of free span at one time. To further examine the monitoring system, two free spans were created (6 m and 2.5 m). It can be seen from Figure 13(d) that the excess temperature curves show two troughs, each representing the relevant free span. Table 4 shows the comparison of simulated and detected free span length, and their error analysis. As previously mentioned, considering the subsea pipelines are over hundreds of kilometers, this level of deviation is negligible. The two features were also calculated, as shown in Figures 14, 15 and 16, respectively.

### 5.3. Differences between Constant-temperature and Constant-power Thermal Cables

**Figure 17.**
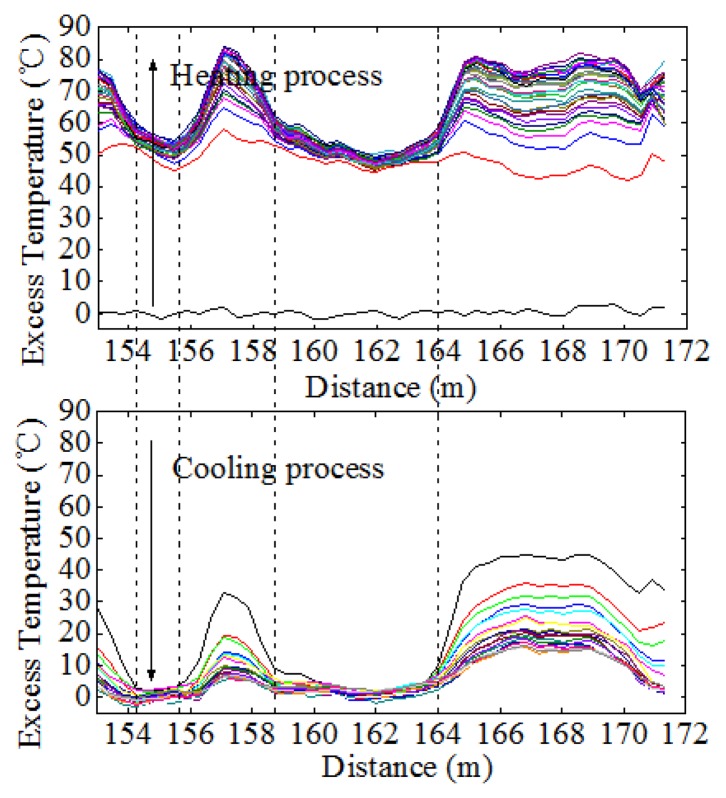
Excess temperature curves of Sensor 3 under two free spans condition.

Figure 17 shows the excess temperature curves obtained from Sensor 3 under two free span conditions. The overall excess temperature in heating process was much higher from Sensor 1 and Sensor 2 due to the higher power input of the constant-temperature heating belt and its unique heating mechanism. Higher excess temperature does not seem to provide higher resolution compared to the constant-power heating belt. After heating for 3 hours, the highest temperature within Sensor 1 and Sensor 3 were 31 °C and 84 °C. However, temperature gap between in-sand and in-water scenarios showed no significant difference as the gap for Sensor 1 was approximately 20 °C and the gap for Sensor 3 was 25 °C. The constant-temperature heating belt was powered by a direct alternating current. The active operating time and maximum length of the belt are limited. However, as the power was supplied by the explosion-proof temperature controller, the constant-power heating belt can work continuously with low energy consumption. Furthermore, the heating belt can be combined to synthesize several kilometers in a strand. In practice, for the constant-power heating belt is preferable for its controllable surface temperature and lower working power input in long distance subsea pipeline scour monitoring.

### 5.4. Validating with Numerical Studies

**Figure 18.**
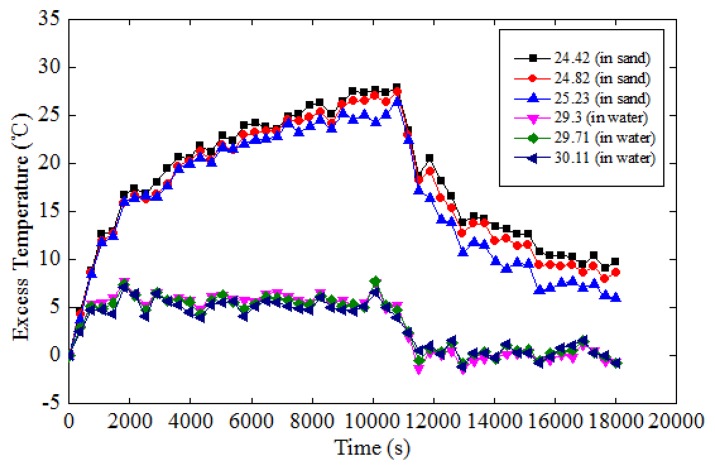
Excess temperature as a function of time for in-sand and in-water scenarios.

In the case of free span with a length of 6 m, the shape of the temperature curves as a function of distance for numerical study and experimental studies exhibits similar behavior, as shown in Figure 3 and Figure 13(c), respectively. By picking out some sampling points from in-water and in-sand scenarios, excess temperature as a function of time was acquired, as shown in Figure 18. Figure 18 shows that for both heating and cooling processes, the excess temperature in the sand and in the water has a logarithmic and exponential relationship with time, respectively. In the sand, temperature increased according to expression (In*t* + In 4*α*/*r*^2^ − *γ*) · *q*/(4*πλ*) during the heating process, and decreased according to In (*t*/(*t* − *t*_1_)) · *q*/4*πλ* [Equation (3)] during the cooling process. In the water, it exponentially reached a plateau (*q*/*hA*) after heating started, and exponentially declined asymptotically after heat stopped [Equation (5)]. For most of the possible thermal parameters in subsea environment, (In*t* + In 4*α*/*r*^2^ − *γ*) · *q*/(4*πλ*) will exceed *q*/*hA* in several seconds, and In (*t*/(*t* − *t*_1_)) · *q*/(4*πλ*) is larger than zero. Thus, the average excess temperature in the sand is higher than that in the water. In addition, the experiments were performed in a running water flow environment, resulting in a larger convective film coefficient. Thus, the excess temperature gap between in-water and in-sand scenarios will be even greater. In general, the curves in Figure 4 and Figure 18 show similar behavior. However, there exist some differences. In experimental studies, temperature curves of the in-water scenario during the heating process became horizontal lines after several minutes. In numerical study, they increased at a slower slope. This is because the water in the numerical study was assumed to be stagnant, causing the heat to accumulate in the water. While in experimental studies, the water flowed in a running cycle, so the heat was swiftly transported away through the water flow and finally reached a thermal balance. The experimental studies show reasonable agreement with the numerical simulation, which further confirm the feasibility of the proposed method in monitoring subsea pipeline scour.

### 5.5. Discussion

These results demonstrate that the proposed active thermometry-based scour monitoring system is effective and reliable for scour monitoring of subsea pipeline systems. The method proved sensitive to both the exposure problem and the free span problem of subsea pipeline, and is capable of locating and identifying the exposure condition and free span length of the pipeline. While the laboratory results are encouraging, field application of the proposed method to monitor scour of subsea pipeline are expected to bring additional sources of uncertainty. Expected primary sources of departure include poor contact between thermal cable and sediment and the spatial variability of the sediment thermal properties along the pipeline.

This system leaves much scope for further development. A signal processing system has the function of diagnosis and auto-alarm needs to be developed to analyze the data from the BOTDA. If the detected free span length reaches the allowable length, the system will send out a warning and concurrently give the precise free spanning location, so that the operator can take preventive measures in time to avoid the failure of the pipeline. Once the exposure and free spanning condition of the pipeline is obtained, the system will access the health status along the pipeline by adopting elasticity mechanics and give the damage factors along the pipeline. At the same time, a more efficient algorithm should be created to identify the sand and water ambient after heating for several minutes, rather than several hours.

## 6. Conclusions

On account of the different heat transfer behaviors between in-sand and in-water scenarios, a scour monitoring system for subsea pipeline was developed aimed at achieving real-time scour monitoring of subsea pipeline. The proposed monitoring system was based on active thermometry and distributed Brillouin optical fiber sensing techniques. By coupling the distributed optical fiber sensors with a heating belt, the thermal cable was utilized to distinguish the ambient environment along the pipeline. Through numerical study of heat transfer behaviors of a line source that was placed in the vicinity of pipeline system, the active thermometry method was verified to be capable of distinguishing the in-sand and in-water scenarios and detecting the scour condition of the pipeline. The exposure and free span tests of a scaled subsea pipeline system was designed and accomplished in laboratory. The experimental results show good agreement with the numerical study and provided promising results for the detection and localization of the exposure and free span condition of subsea pipeline. When it comes to field application, the constant-power heating belt is preferred for its low power consumption and high performance. The scour monitoring system provides many advantages such as high sensitivity and precision, low cost, and construction flexibility.

## Figures and Tables

**Table 1.** Material parameters used in simulation.

**Material**	**Conductivity** **(W/(m · °C))**	**Density** **(kg/*m*^3^)**	**Specific heat** **(J/(kg · °C))**
Water	0.6	1000	4200
Sand	3	1922	830
Copper	401	8930	385

**Table 2.** Specifications for the BOTDA analyzer.

**Parameter**	**Value**
Spatial resolution	1.0 m
Sampling interval	0.41 m
Central frequency	10.9255 GHz
Frequency step	0.003 GHz
Sampling time interval	6 min

**Table 3.** Comparison of simulated and detected exposure length from Sensor 2.

**Exposure Length (m)**	**Emerged Section**		**Detected Length (m)**	**Error (m)**	**Error Rate (%)**

	**From (m)**	**To (m)**			
2.0	96.04	97.26	2.22	0.22	11.00
4.0	96.04	98.89	3.85	−0.15	3.75
5.2	95.63	99.70	5.07	−0.13	2.50

**Table 4.** Comparison of simulated and detected free span length.

**Free Span Length (m)**		**Emerged Section**		**Detected Length (m)**	**Error (m)**	**Error rate (%)**

		**From (m)**	**To (m)**			
Sensor 1	2.0	27.27	28.08	1.81	−0.19	9.50
	2.5	21.98	23.20	2.22	−0.28	11.20
	4.0	27.27	30.11	3.84	−0.16	4.00
	6.0	26.04	30.93	5.89	−0.11	1.83

Sensor 2	2.0	96.04	96.85	1.81	−0.19	9.50
	2.5	90.75	92.38	2.63	0.13	5.20
	4.0	96.04	99.30	4.26	0.26	6.50
	6.0	95.23	100.11	5.88	−0.12	2.00

Sensor 3	2.0	160.34	161.15	1.81	−0.19	9.50
	2.5	154.23	155.86	2.63	0.13	5.20
	4.0	159.93	162.78	3.85	−0.15	3.75
	6.0	158.71	163.59	5.88	−0.12	2.00

## References

[1] Drago M., Pigliapoco M., Ciuffardi T. Analysis of Pipeline Fatigue Damage for Scour Induced Freespans.

[2] Herbich J.B. (1981). Offshore Pipeline Design Elements.

[3] Jin W.L., Zhang E.Y., Shao J.W., Liu D.H. (2004). Cause analysis and countermeasure for submarine pipeline failure. Bull. Sci. Tech..

[4] Jin W.L., Shao J.W., Zhang E.Y. Basic Strategy of Health Monitoring on Submarine Pipeline by Distributed Optical Fiber Sensor.

[5] Jin W.L., Shao J.W. A Practical Algorithms on Health Monitoring of Submarine Pipeline and Its Application.

[6] Bao C.X., Hao H., Li Z.X. (2012). Vibration-based structural health monitoring of offshore pipelines: numerical and experimental study. Struct. Control Health Monit..

[7] Campbell G.S., Calissendorff C., Williams J.H. (1991). Probe for measuring soil specific heat using a heat-pulse method. Soil Sci. Soc. Am. J..

[8] Bristow K.L., Campbell G.S., Calissendorff K. (1993). Test of a heat-pulse probe for measuring changes in soil water content. Soil Sci. Soc. Am. J..

[9] Krishnaiah S., Singh D.N., Jadhav G.N. (2004). A methodology for determining thermal properties of rocks. Int. J. Rock Mech. Mining.

[10] Bristow K.L. (1998). Measurement of thermal properties and water content of unsaturated sandy soil using dual-probe heat-pulse probes. Agric. For. Meteorol..

[11] Freifeld B.M., Finsterle S., Onstott T.C., Toole P., Pratt L.M. (2008). Ground surface temperature reconstructions: using *in situ* estimates for thermal conductivity acquired with a fiber-optic distributed thermal perturbation sensor. Geophys. Res. Lett..

[12] Cote A., Carrier B., Leduc J., Noel P., Beauchemin R., Soares M., Garneau C., Gervais R. Water Leakage Detection Using Optical Fiber at the Peribonka Dam.

[13] Sayde C., Gregory C., Gil-Rodriguez M., Tufillaro N., Tyler S., van de Giesen N., English M., Cuenca R., Selker J.S. (2010). Feasibility of soil moisture monitoring with heated fiber optics. Water Resour. Res..

[14] Zhao X.F., Li L., Ba Q., Ou J.P. (2012). Scour monitoring system of subsea pipeline using distributed Brillouin optical sensors based on active thermometry. Optical Laser Tech..

[15] Gong Y.D. (2007). Guideline for the design of a fiber optic distributed temperature and strain sensor. Optics Commun..

[16] de Vries D.A., Peck A.J. (1958). On the cylindrical probe method of measuring thermal conductivity with special reference to soils. I extension of theory and discussion of probe characteristics. Aust. J. Phys..

